# Effectiveness of Artificial Intelligence in Endodontic Diagnosis and Treatment Evaluation: A Systematic Review

**DOI:** 10.7759/cureus.96091

**Published:** 2025-11-04

**Authors:** Mazen Doumani, Fatmah Almaqboul, Sultan Saad S Alduwaysan, Mohammed A Alzahrani, Saud A Al Ghamdi, Mohammed N Alzahrani, Alwaleed T Alanazi, Nawaf M Al Ghamdi, Abdulrahman H Alsalem, Dalal Alturaif, Ferial H Almokayad, Abeer S Alqahtani, Laila Alanaz

**Affiliations:** 1 Department of Conservative Dentistry, Alfarabi College of Dentistry and Nursing, Riyadh, SAU; 2 General Dentistry, Ministry of Health, Al Baha, SAU; 3 Dentistry, King Saud University, Riyadh, SAU; 4 Dentistry, Al Baha University, Al Baha, SAU; 5 Dentistry, Taif University, Al-Hawiyya, SAU; 6 Dentistry, Riyadh Elm University, Riyadh, SAU; 7 Dentistry, King Khalid University Hospital, Abha, SAU; 8 Restorative Dentistry, Private Practice, Riyadh, SAU; 9 Dentistry, Almustaqbal University, Hillah, IRQ; 10 Dentistry, King Khalid University, Riyadh, SAU; 11 Dentistry, Private Practice, Riyadh, SAU

**Keywords:** artificial intelligence, cbct, deep learning, dental imaging, endodontics, root canal

## Abstract

Artificial intelligence (AI) has emerged as a transformative tool in endodontics, offering potential to enhance diagnostic accuracy, treatment evaluation, and clinical decision-making. This systematic review aimed to assess the effectiveness of AI models in diagnosing endodontic conditions and evaluating treatment outcomes compared with human clinicians. Following Preferred Reporting Items for Systematic Reviews and Meta-Analyses (PRISMA) 2020 guidelines, a comprehensive literature search was conducted across PubMed, ScienceDirect, Web of Science, Cochrane Library, and Google Scholar up to June 2025. Ten studies met the inclusion criteria, encompassing diverse AI models such as convolutional neural networks (CNNs), U-Net architectures, YOLOv5, and ChatGPT-4. These were applied to tasks including periapical lesion detection, root canal filling evaluation, fractured instrument identification, and caries diagnosis. Across studies, AI systems achieved diagnostic accuracy ranging from 75% to 99%, with sensitivity and specificity frequently exceeding 80%. DenseNet201 achieved the highest performance for fractured instrument detection (area under the curve (AUC) = 0.900; Matthews correlation coefficient (MCC) = 0.810), while ChatGPT-4 demonstrated superior diagnostic accuracy (99%) compared with dental students (77-80%). Cone-beam computed tomography (CBCT)-based models consistently outperformed those using panoramic or periapical images. Despite high accuracy, variability in methodologies, dataset sizes, and outcome metrics limited quantitative synthesis; therefore, a meta-analysis was not feasible. Overall, AI demonstrated comparable or superior performance to clinicians, offering advantages in speed, reproducibility, and objectivity. However, limitations such as dataset heterogeneity, lack of external validation, and challenges in detecting subtle features underscore the need for further large-scale, multicenter studies. AI integration into routine endodontic practice shows strong potential but requires continued refinement and validation for reliable clinical adoption.

## Introduction and background

Endodontic therapy aims to preserve the natural tooth by eliminating infection, maintaining peri-radicular tissue health, and restoring function. Despite advances in materials, instrumentation, and imaging technologies, failure rates in general dental practice persist at 15-40% due to diagnostic inaccuracies, anatomical complexities, and treatment variability [1,2]. Some studies even report failure rates as much as around 80% [3]. Achieving optimal outcomes hinges on accurate diagnosis, effective decision-making, and precise treatment planning - all of which remain elusive in many clinical scenarios.

Artificial intelligence (AI), particularly deep learning approaches such as convolutional neural networks (CNNs), has revolutionized medical image analysis across multiple specialties due to its capacity for high-throughput, reproducible detection of pathological features [4]. In dentistry, and especially in endodontics, AI has been applied to various tasks including working-length determination, detection of periapical pathoses and vertical root fractures, and evaluation of root canal morphology and treatment outcomes [4,5]. U-Net architectures, You Only Look Once (YOLO)-based detectors, and CNN ensembles have shown high accuracy in identifying periapical lesions on radiographs, with sensitivity and specificity often surpassing 0.80-0.90 [6].

A systematic review by Boreak et al. summarized 10 studies that utilized CNNs and artificial neural networks (ANNs) for tasks such as apical foramen localization and retreatment outcome prediction, concluding that AI matched - or in some cases exceeded - specialist performance [4]. More recent scoping reviews and meta-analyses confirm that AI platforms offer diagnostic accuracy comparable to experts, with added benefits of speed and operational efficiency [5]. Nevertheless, challenges persist, including inconsistency in dataset quality, limited external validation, and the need for explainable, clinically integrated models [7]. Despite promise, the clinical adoption of AI in endodontics remains limited. Standardized evaluation protocols, multi-center trials, and real-world validation across diverse populations are essential to substantiate AI’s reliability and foster trust among clinicians. This systematic review addresses these gaps by evaluating current evidence on AI effectiveness in endodontic diagnosis and treatment assessment, with emphasis on performance relative to human experts, diagnostic metrics, imaging modality, and study design quality.

## Review

Methodology

Study Design

This systematic review was conducted in accordance with the Preferred Reporting Items for Systematic Reviews and Meta-Analyses (PRISMA) 2020 guidelines. This systematic review was not registered in PROSPERO as it primarily involved qualitative synthesis of diagnostic accuracy studies and did not include intervention-based data. The objective of the review is to evaluate the effectiveness of AI in the diagnosis and treatment of endodontic conditions. The review included qualitative syntheses to provide a comprehensive summary of current evidence.

Eligibility Criteria

The review included original studies involving human subjects where AI-based technologies have been applied in endodontic diagnosis or treatment. Eligible studies included randomized controlled trials (RCTs), cohort studies, case-control studies, cross-sectional studies, and observational designs that compare AI-assisted methods with conventional diagnostic or treatment approaches. Studies were included if they reported clinical outcomes such as diagnostic accuracy, treatment success rates, time efficiency, or patient satisfaction. Only studies published in English and from inception till June 2025 were considered. Only English-language studies were included due to resource limitations and to ensure accurate data interpretation. Exclusion criteria included non-human or in vitro studies, review articles, editorials, commentaries, conference abstracts without full text, and studies not reporting relevant clinical outcomes.

Data Sources

To ensure a comprehensive and unbiased literature search, multiple electronic databases were used. These include PubMed (MEDLINE), ScienceDirect, Web of Science and Cochrane Library, given its relevance to computer science and AI research. Google Scholar was screened for grey literature, and the reference lists of included studies were manually checked to identify further eligible articles.

Search Strategy

A structured and reproducible search strategy was developed using a combination of Medical Subject Headings (MeSH) and free-text terms relevant to artificial intelligence and endodontics. The following keywords were used: "artificial intelligence", "machine learning", "deep learning", "neural networks", "computer-aided diagnosis", "endodontics", "root canal", "pulpitis", and "periapical lesions". Boolean operators (AND/OR) were used to combine the terms appropriately. The literature search was conducted between April 1, 2025, and June 10, 2025, across the specified databases.

Study Selection

**Figure 1:**
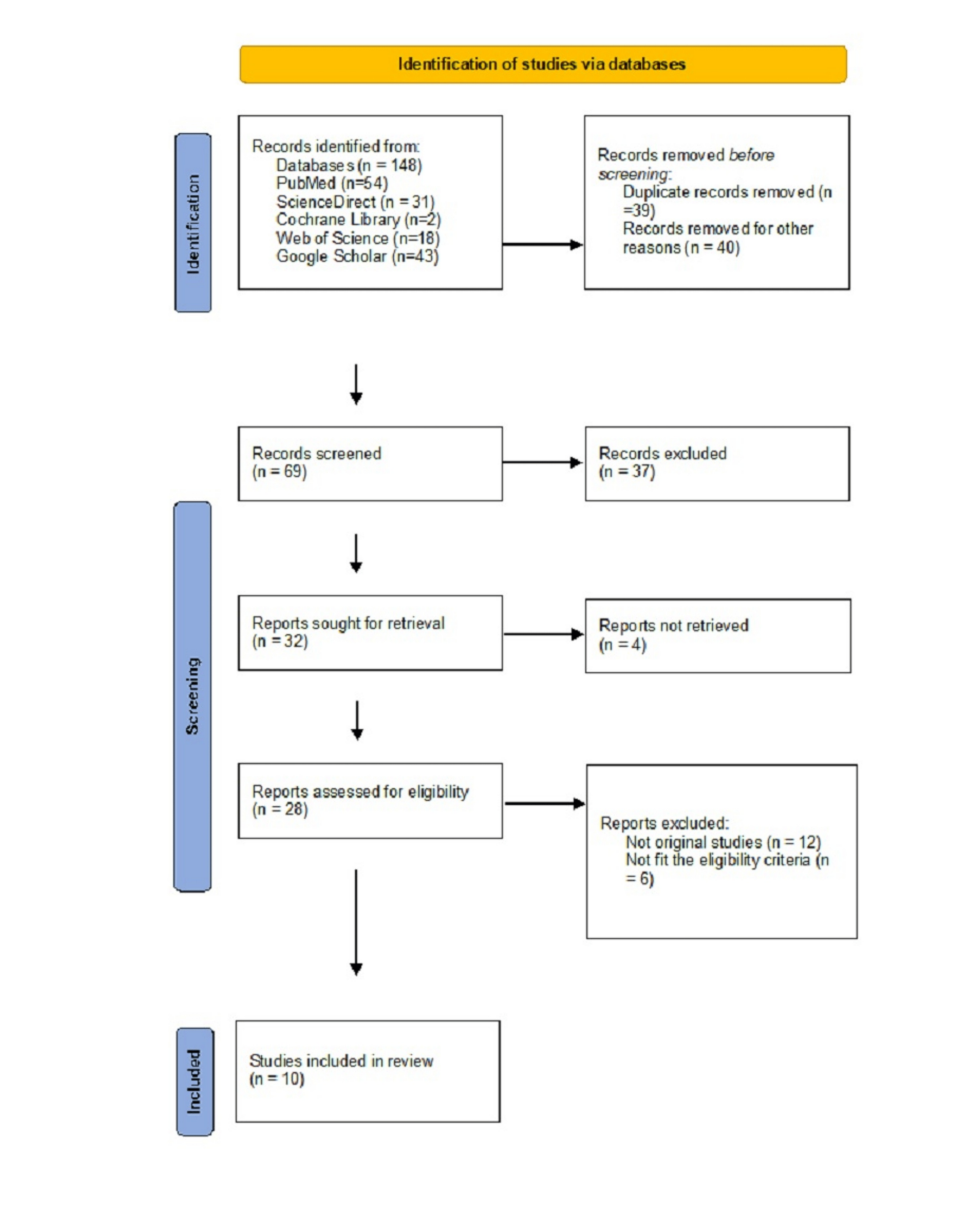
Preferred Reporting Items for Systematic Reviews and Meta-Analyses (PRISMA) flowchart showing the study selection process.

All search results were imported into reference management software to remove duplicates. The selection process followed two stages. In the first stage, two reviewers independently screened titles and abstracts to identify potentially relevant studies. In the second stage, full texts of shortlisted articles were retrieved and assessed for eligibility based on the inclusion and exclusion criteria. Any disagreements between reviewers were resolved through discussion or, if needed, by consulting a third reviewer. The study selection process is illustrated in Figure 1.

Data Extraction

A standardized data extraction form was developed to ensure consistency across studies. Two reviewers independently extracted data from the included articles. The extracted data included study characteristics (such as author, publication year, country, and study design), population details (sample size and demographics), the type of AI model used, the nature of the comparator (if any), clinical setting, outcomes measured (such as accuracy, sensitivity, specificity, and success rates), and the key findings. Any discrepancies in data extraction were resolved by discussion or adjudication by a third reviewer.

Meta-Analysis

A quantitative meta-analysis was not performed due to substantial heterogeneity among the included studies. The selected studies varied considerably in their AI models (e.g., CNNs, U-Net, YOLOv5, ChatGPT-4), imaging modalities (periapical radiographs, panoramic radiographs, cone-beam computed tomography (CBCT), intraoral images), study designs (retrospective, prospective, diagnostic accuracy, and comparative evaluations), and outcome measures (accuracy, sensitivity, specificity, F1 score, area under the curve (AUC), Dice coefficient). These methodological and clinical differences precluded meaningful statistical pooling of data. Therefore, a qualitative synthesis was undertaken to summarize the diagnostic performance and clinical effectiveness of artificial intelligence applications in endodontics.

Quality Assessment

The methodological quality of the included studies was evaluated using a modified version of the Newcastle-Ottawa Scale (NOS) adapted for diagnostic accuracy research [8,9]. Two reviewers independently assessed the risk of bias in three domains: selection, comparability, and outcome assessment. Discrepancies were resolved through discussion. 

Results

A total of 148 records were identified through database searches, including PubMed (n = 54), ScienceDirect (n = 31), Cochrane Library (n = 2), Web of Science (n = 18), and Google Scholar (n = 43). After removing 39 duplicates and 40 records for other reasons (e.g., irrelevant or incomplete data), 69 records were screened based on title and abstract. Of these, 37 were excluded for not meeting the inclusion criteria. The full texts of 32 articles were retrieved, and four could not be accessed. Following full-text assessment, 18 studies were excluded for reasons such as being review articles (n = 12) or lacking clinical or diagnostic outcome data (n = 6). Ultimately, 10 studies met the eligibility criteria and were included in the qualitative synthesis. Only studies with moderate to high quality were included in the systematic review. The results of quality assessment are shown in Table 1.

**Table 1:** Risk of Bias Assessment of Included Studies (Modified Newcastle–Ottawa Scale) *Quality Rating: 7–9 = High, 4–6 = Moderate, 0–3 = Low quality [8]

Study (Author, Year)	Selection (0–4)	Comparability (0–2)	Outcome/Assessment (0–3)	Total Score (0–9)	Quality Rating*
Allihaibi et al. (2025) [10]	4	2	2	8	High
Çatmabacak & Çetinkaya (2025) [6]	4	1	2	7	Moderate
Issa et al. (2023) [11]	3	2	2	7	Moderate
Jin et al. (2025) [12]	4	2	3	9	High
Kazimierczak et al. (2024, PAN) [13]	3	1	2	6	Moderate
Kazimierczak et al. (2024, CBCT) [14]	4	1	2	7	Moderate
Qutieshat et al. (2024) [15]	3	2	2	7	Moderate
Zhang et al. (2024) [16]	4	2	3	9	High
Boztuna et al. (2024) [17]	3	1	2	6	Moderate
Chau et al. (2025) [18]	4	2	3	9	High

These studies encompassed a variety of AI models, including CNNs, deep learning architectures (e.g., DenseNet201, ResNet-18, EfficientNet B0, VGG-19, MaxVit-T), and natural language processing tools (e.g., ChatGPT-4). The studies were conducted across diverse geographical regions, including the UK, Saudi Arabia, Turkey, Poland, China, and Oman, and employed various study designs such as retrospective analyses, diagnostic accuracy comparisons, and prospective clinical evaluations. This is summarized in Table 2.

**Table 2:** Summary Characteristics of Included Studies CNN: convolutional neural network, CBCT: cone-beam computed tomography, DL: deep learning, PA: periapical radiograph, FEI: fractured endodontic instrument, AUC: area under the curve, MCC: Matthews correlation coefficient, PAI: periapical index, PAN: panoramic, IoU: Intersection over Union, NPV: negative predictive value, PPV: positive predictive value, PPR: progressive prediction refinement

Study (Author, Year)	Country	Study Design	Sample Size	AI Model Used	Comparator	Clinical Outcome(s)	Key Findings
Allihaibi et al. (2025) [10]	UK/Saudi Arabia	Retrospective diagnostic accuracy	376 teeth (860 roots)	Diagnocat (CNN-based)	Expert endodontists (CBCT as reference)	Sensitivity, specificity, accuracy at tooth/root level	AI sensitivity: 67.3% (tooth), 54.3% (root); specificity: 82.3% (tooth), 86.7% (root). Comparable accuracy to clinicians (76.3% vs. 75.3%).
Çatmabacak & Çetinkaya (2025) [6]	Turkey	Diagnostic accuracy (DL model comparison)	700 PAs (381 teeth with FEIs)	DenseNet201, ResNet-18, EfficientNet B0, VGG-19, MaxVit-T	Ground-truth annotations	Accuracy, AUC, MCC for FEI detection	DenseNet201 outperformed others (AUC: 90.0%, MCC: 81.0%). MaxVit-T underperformed (AUC: 56.0%).
Issa et al. (2023) [11]	Poland	Retrospective diagnostic accuracy	20 PAs (60 teeth)	Diagnocat (U-Net-like)	Expert radiologists (PAI index)	Sensitivity, specificity, F1 score	High accuracy (96.66%), sensitivity (92.30%), specificity (97.87%). 1 FP/FN.
Jin et al. (2025) [12]	China	Diagnostic accuracy	1,508 radiographs	YOLOv5	Inexperienced endodontist	Evaluation of root-canal filling quality (correct/incorrect) on periapical radiographs	YOLOv5 outperformed inexperienced endodontists (F1 scores: 92.05% correct, 82.93% incorrect). Evaluated images 150–220x faster than manual methods.
Kazimierczak et al. (2024) [13]	Poland	Diagnostic accuracy	55 PAN images	Diagnocat (CNN)	Human readers' consensus	Filling probability, obturation adequacy, density, overfilling, voids, short filling	High accuracy for filling probability (90.72%), but lower performance for voids (F1=14.29%) and short fillings (F1=8.33%). PAN limitations noted.
Kazimierczak et al. (2024) [14]	Poland	Diagnostic accuracy	55 CBCT images	Diagnocat (CNN)	Human readers' consensus	Filling probability, obturation adequacy, density, overfilling, voids, short filling	High accuracy for most parameters (e.g., 100% for filling probability, 95.5% for overfilling). Lower recall for voids (66.7%). CBCT superior to PAN for endodontic assessment.
Qutieshat et al. (2024) [15]	Oman	Comparative diagnostic accuracy	109 dental students	Modified ChatGPT-4	Dental students (juniors/seniors)	Pulpal and apical diagnoses in endodontic assessments	ChatGPT achieved significantly higher accuracy (99.0%) vs. seniors (79.7%) and juniors (77.0%). Highlights AI's potential as a reference tool in dental education.
Zhang et al. (2024) [16]	China	Prospective clinical evaluation	4,361 teeth (191 patients)	MobileNet-v3 + U-net	Clinical diagnosis by specialists	Caries detection via intraoral images	Overall accuracy: 93.40%; sensitivity: 81.31%, specificity: 95.65%. High NPV (96.49%) but variable PPV (77.68%). Best performance in anterior teeth (96.04% accuracy).
Boztuna et al. (2024) [17]	Cyprus	Retrospective diagnostic study	400 panoramic radiographs; 780 periapical lesions	U²-Net (deep learning, segmentation model)	Manual annotation by experienced clinicians	Dice coefficient, IoU, Precision, Recall, F1-score	Dice = 78.8%; IoU = 71.5%; Precision = 77.6%; Recall = 85.4%; F1 = 81.0% on test set. Model evaluated a radiograph in ~1.2s. High agreement with human annotation.
Chau et al. (2025) [18]	China	Diagnostic performance evaluation	185 CBCT scans	CBCT-SAM (with and without PPR), Modified U-Net, PAL-Net	Manual segmentation validated by expert	Diagnostic accuracy, segmentation accuracy, sensitivity, specificity, precision, Dice Similarity Coefficient (DSC)	CBCT-SAM achieved 98.92% diagnostic accuracy, 99.65% segmentation accuracy; sensitivity 72.36%, specificity 99.87%, DSC 70.0%. Outperformed Modified U-Net; performance comparable to PAL-Net.

Diagnostic Accuracy of AI in Endodontics

The included studies demonstrated that AI achieves high diagnostic accuracy in detecting various endodontic conditions. Allihaibi et al. [10] reported an AI sensitivity of 67.3% at the tooth level and 54.3% at the root level, with specificity values of 82.3% and 86.7%, respectively. These results were comparable to those of expert endodontists, who achieved an accuracy of 76.3% versus AI’s 75.3%. Çatmabacak and Çetinkaya [6] evaluated multiple deep learning models and found that DenseNet201 performed exceptionally well in detecting furcation involvement, with an AUC of 0.900 and an MCC of 0.810, while MaxVit-T underperformed with an AUC of 0.560. Issa et al. [11] utilized a U-Net-like model for periapical lesion detection and reported high accuracy (96.66%), sensitivity (92.30%), and specificity (97.87%). Zhang et al. [16] focused on caries detection using intraoral images and achieved an overall accuracy of 93.40%, with particularly strong performance in anterior teeth (96.04% accuracy). Boztuna et al. [17] evaluated the performance of a U²-Net deep learning model for detecting periapical lesions on 400 panoramic radiographs, comprising 780 manually annotated lesions. The model achieved a Dice coefficient of 0.788, an Intersection over Union (IoU) of 71.5%, precision of 77.6%, recall of 85.4%, and an F1-score of 81.0% on the test set. The AI system processed each radiograph in approximately 1.2 seconds, demonstrating robust diagnostic capability and efficiency.

AI in Evaluating Endodontic Treatment Outcomes

AI models were also effective in assessing the quality of endodontic treatments. Jin et al. [12] demonstrated that the YOLOv5 model outperformed inexperienced endodontists in evaluating root canal filling quality, achieving F1 scores of 92.05% for correct fillings and 82.93% for incorrect fillings. Additionally, the AI system processed images 150-220 times faster than manual methods. Kazimierczak et al. [13,14] examined AI’s ability to assess root canal obturation and found high accuracy for filling probability (90.72% in PAN images, 100% in CBCT images). However, the model struggled with detecting voids (F1 score: 14.29%) and short fillings (F1 score: 8.33%). The study also noted that CBCT-based AI assessments were superior to PAN for most parameters, such as overfilling detection (95.5% accuracy). In a comparative diagnostic study, Qutieshat et al. [15] evaluated the performance of ChatGPT-4 against dental students in diagnosing pulpal and apical conditions. The AI system achieved a significantly higher accuracy rate (99.0%) compared to senior dental students (79.7%) and junior students (77.0%). This highlights AI’s potential as a supplementary tool in dental education and clinical decision-making. Chau et al. [18] introduced CBCT-SAM, a novel deep learning model designed to detect periapical lesions on CBCT scans. Using 185 annotated CBCT images, the model achieved an average diagnostic accuracy of 98.92% and segmentation accuracy of 99.65%. Sensitivity, specificity, and Dice Similarity Coefficient (DSC) were 72.36%, 99.87%, and 0.70, respectively. CBCT-SAM outperformed the Modified U-Net and showed comparable performance to PAL-Net, demonstrating expert-level capability in identifying periapical lesions and potential for chairside clinical use.

Discussion

The findings of this systematic review demonstrate that AI holds significant promise in enhancing diagnostic accuracy and treatment evaluation in endodontics. AI models, particularly deep learning-based systems such as CNNs and U-Net architectures, consistently achieved performance levels comparable to or exceeding those of human clinicians in tasks like detecting periapical lesions, assessing root canal filling quality, and diagnosing caries. Recent review by Karobari et al. [19], corroborate these findings, showing that convolutional neural networks outperform conventional radiographic assessments in detecting periapical and pulpal pathoses while identifying similar limitations in 2D imaging and external validation gaps. For instance, DenseNet201 demonstrated exceptional performance in detecting fractured instruments, with an AUC of 0.900 and an MCC of 0.810, outperforming other models such as MaxVit-T (AUC: 0.560) [6]. Similarly, YOLOv5 significantly surpassed inexperienced clinicians in evaluating root canal fillings, processing images 150-220 times faster than manual methods [12]. These results align with previous research indicating that AI can reduce subjectivity in radiographic interpretation, minimize diagnostic errors, and improve workflow efficiency [7].

Recent evidence also supports that AI’s diagnostic sensitivity may at times exceed that of clinicians, but with trade-offs in specificity. Allihaibi et al. (2025) demonstrated that an AI-based platform achieved high sensitivity but slightly lower specificity compared to human evaluators when CBCT was used as the reference standard, emphasizing that AI should complement, not replace, clinical judgment [10]. A systematic review by Naik et al. [20] found that AI tools outperformed clinicians in detecting periapical lesions with sensitivity and specificity ranging from 65-97% and 83-97% respectively. Similarly, Choudhari et al. [21] systematically reviewed AI versus human experts in predicting endodontic outcomes, finding AI models to outperform or match experts across tasks like working length determination and lesion detection. More recently, Kot et al. [22] performed a meta-analysis of deep learning segmentation methods on CBCT and CT datasets, confirming their high accuracy but also highlighting significant heterogeneity across datasets and annotation protocols, reinforcing the need for standardization in future endodontic AI research.

Furthermore, Thurzo et al. highlighted the role of AI in identifying root fractures and optimizing treatment planning, which underscores its importance in minimizing failures in endodontic therapy and enhancing the success rate of treatments [23]. An international study using CNN and segmentation models on panoramic radiographs reported AI sensitivity of 67.9% for periapical radiolucency and AUC of 96.2%, performance on par with clinicians and at much faster speeds [24]. These findings align with multi-center studies on federated learning (FL), which demonstrate that distributed AI models can achieve near-equivalent accuracy to centralized systems while preserving patient data privacy - a critical factor for multi-institutional collaborations in dentistry [25]. Moreover, recent work showed that FL remains robust against annotation errors and image noise, indicating its suitability for real-world, heterogeneous datasets [26].

In this review, the included studies covered a wide range of AI applications, imaging modalities, and clinical tasks, all of which underscore the expanding utility of AI in endodontic practice. For example, Boztuna et al. [17] employed a U²-Net segmentation model to detect periapical lesions on 400 panoramic radiographs and reported strong diagnostic performance (F1-score = 0.81, Dice coefficient = 0.788), demonstrating the model’s potential as a fast and accurate diagnostic aid. Similarly, Chau et al. [18] introduced CBCT-SAM, a novel model developed specifically for CBCT imaging, which achieved a remarkable diagnostic accuracy of 98.92% and segmentation accuracy of 99.65%, significantly outperforming Modified U-Net and performing comparably to PAL-Net. These results suggest that CBCT-based AI, when paired with dedicated model architectures like CBCT-SAM, can deliver expert-level performance in detecting periapical pathology.

Furthermore, the diversity of AI models used in the included studies - ranging from DenseNet201 for fractured instrument detection, YOLOv5 for root canal filling evaluation, and ChatGPT-4 for diagnostic support - highlights the flexibility and adaptability of AI technologies across both image-based and text-based diagnostic tasks. Notably, several models performed significantly better than novice or intermediate human evaluators, as seen in the studies by Jin et al. [12] and Qutieshat et al. [15], which not only supports the diagnostic value of AI but also points toward its potential use in dental education and decision support.

While this review highlights the promising role of AI in endodontics, several limitations must be acknowledged. First, the included studies exhibited heterogeneity in dataset sizes, imaging modalities (e.g., CBCT vs. panoramic radiographs), and outcome metrics, complicating direct comparisons. These challenges are consistent with recent large-scale analyses emphasizing the variability in labeling quality and imaging conditions across datasets, which can significantly affect AI reliability [22,26]. Second, most AI models were trained and validated on retrospective, single-center datasets, raising concerns about generalizability to diverse populations and clinical settings. Third, external validation was scarce, with only one study testing AI performance on independent datasets; thus, real-world applicability remains uncertain. Additionally, AI struggled with subtle features (e.g., voids, short fillings) due to resolution constraints, particularly in 2D radiographs. Moreover, Language restriction to English publications may have introduced selection bias by excluding relevant non-English studies. Finally, the lack of standardized protocols for AI integration into clinical workflows and the absence of long-term outcome data limit conclusions about sustained effectiveness. Future work should prioritize external validation across diverse populations and imaging modalities, incorporation of explainability, exploring multimodal data (clinical + radiographic + patient history), and ensuring models are robust to labeling errors and image noise. Studies such as the 2025 FL segmentation resilience work offer valuable blueprints for how to address the latter two [26]. Furthermore, future studies should prioritize multi-center collaborations, prospective trials, and explainable AI frameworks to address these gaps.

## Conclusions

The findings of this systematic review demonstrate that AI has substantial potential to enhance diagnostic accuracy and treatment evaluation in endodontics. AI models, particularly deep learning-based systems, consistently achieved performance levels comparable to or exceeding those of human clinicians in tasks such as detecting periapical lesions, assessing root canal filling quality, and diagnosing caries. Notably, AI systems demonstrated advantages in processing speed and consistency, reducing subjectivity inherent in manual evaluations. However, the review also identified key limitations, including variability in AI performance across different tasks, challenges in detecting subtle endodontic features (e.g., voids or short fillings), and reliance on limited datasets in some studies. These factors highlight the need for further refinement of AI algorithms and validation in diverse clinical settings. Future research should focus on expanding dataset sizes, improving model generalizability, and integrating AI tools into real-world clinical workflows. Additionally, interdisciplinary collaboration between endodontists and AI developers will be crucial to address current limitations and optimize these technologies for practical use.
